# *In vivo* analgesic, anti-inflammatory and molecular docking studies of *S*-naproxen derivatives

**DOI:** 10.1016/j.heliyon.2024.e24267

**Published:** 2024-01-10

**Authors:** Naveed Muhammad, Rashid Khan, Faiza Seraj, Abad Khan, Ubaid Ullah, Abdul Wadood, Amar Ajmal, Uzma, Basharat Ali, Khalid Mohammed Khan, Noor Ul Ain Nawaz, Najla AlMasoud, Taghrid S. Alomar, Abdur Rauf

**Affiliations:** a Department of Pharmacy, Abdul Wali Khan University, Mardan, Pakhtunkhwa, Pakistan; b Department of Pharmacy, University of Swabi, Swabi, Khyber Pakhtunkhwa, Pakistan; c H. E. J. Research Institute of Chemistry, International Center for Chemical and Biological Sciences, University of Karachi, Karachi, 75270, Pakistan; d Department of Biochemistry, Abdul Wali Khan University, Mardan, Pakhtunkhwa, Pakistan; e Sulaiman Bin Abdullah Aba Al-Khail (SA)- Center for Interdisciplinary Research in Basic Science, International Islamic University, Islamabad, Pakistan; f Pakistan Academy of Science, 3-Constitution Avenue, G-5/2, Islamabad, 44000, Pakistan; g Department of Pharmacy, City University of Science and Information Technology Peshawar, Peshawar, Khyber Pakhtunkhwa, Pakistan; h Department of Chemistry, College of Science, Princess Nourah bint Abdulrahman University, P.O. Box 84427, Riyadh, 11671, Saudi Arabia; i Department of Chemistry, University of Swabi, Swabi, 23430, Pakistan

**Keywords:** Naproxen derivatives, Analgesic, Anti-inflammatory, Molecular docking

## Abstract

In the current studies two naproxen derivatives (NPD) were evaluated for analgesic and anti-inflammatory properties. The acetic acid and hot plate animal models were used to screen the compounds for analgesic potential. While the anti-inflammatory potential was evaluated through animal paw edema, induced by several inflammatory mediators (carrageenan, bradykinin, and prostaglandin E2), the xylene-induced ear edema was also used as an inflammatory model. Both NPDs showed significant (p < 0.001) antinociceptive effects in the acetic acid-induced writhing paradigm. In the case of the hot plate, the NPD **1** at the tested dose of 5 mg/kg enhanced the latency time after 60 min of injection, which remained significant (p < 0.001) up to the end of the experiment duration. The maximum percent inhibition of NPD **1** was 87.53. The naloxone injection significantly lowered the latency time of NPD **1** as compared to NPD **2**. Regarding the anti-inflammatory effect, both of the tested NPDs demonstrated a significant reduction in paw edema against various inflammatory mediators, as mentioned above; however, the anti-inflammatory effect of NPD **1** was better. The maximal percent inhibition by NPD **1** and **2** was 43.24 (after 60 min) and 45.93 (after 90 min). A considerable effect also resulted from xylene-induced ere edema. Further, a molecular docking study was carried out to investigate the binding modes of the NPD. The docking analysis revealed that the NPD significantly interacted with the COX2 enzyme. Furthermore, molecular dynamics simulation was carried out for the docked complexes. The MD simulation analysis revealed the high stability of the two naproxen derivatives.

## 1 Introduction

Inflammation was once assumed to be a harmful response to the body, but it was subsequently determined as the body's protective response to the stimuli [1]. Inflammation manifests itself in five ways: pain, swelling, redness, loss of function, and temperature [2]. Some mediators, such as prostaglandins, are synthesized by the cyclooxygenase (COX) pathway and are responsible for inflammation and pain. The COX pathway must be suppressed to prevent or treat pain and inflammation [3]. Non-steroidal anti-inflammatory drugs have recently been the most popular agents to interfere with the COX pathway. These drugs work by antagonizing COX pathways to inhibit the biosynthesis of prostaglandin E1 (PGE1) and prostaglandin E2 (PGE2) [4]. PGE1 benefits several organs, including the kidney and the stomach, whereas PGE2 plays a more prominent role in pathological circumstances [5]. Naproxen is a widely prescribed non-steroidal anti-inflammatory drug (NSAID) that is available over the counter and is used to treat several clinical conditions (migraine, stress-associated headache, pain after surgery, and pain linked with gynecological problems) [6]. Levo (*S*) naproxen has anti-inflammatory and painkiller properties compared to dextro stereo-isomers [7]. Among different NSAIDs, naproxen has a better cardiovascular and gastrointestinal safety profile and no need for dose adjustment in mild hepatic and renal impaired patients [8]. The recommended naproxen dose is 500–1000 mg once or twice daily through oral or rectal route [9]. However, in higher doses, naproxen is notorious for ample adverse drug reactions, especially GIT bleeding and hepatotoxicity. Naproxen 2-(6-methoxy-2-nepthyl) propionic acid is a non-steroidal anti-inflammatory drug (NSAID) having a diverse variety of biologically active derivatives that have been tested for various pharmacological effects. Novel synthesis methods have been achieved to develop naproxen derivatives that have better efficacy, potency, and fewer ADRs [10]. The current studies aimed to test the NPD for analgesic and anti-inflammatory effects with the best hope of finding safe, effective, and economical NPD.

## 2 Material and methods

### 2.1 Chemicals

Diclofenac sodium and naloxone (Suzhou Ausun Chemical Co, Ltd., China), dexamethasone, tramadol (Searle Pakistan Ltd.), carrageenan (Sigma Lambda, USA), prostaglandin, formalin, bradykinin, xylene and dimethyl sulphoxide (Sigma Aldrich, USA), acetic acid (Merck Germany), and distilled water were used as a negative control as well as for the dissolution of test compound and reference.

### 2.2 Animals

For these studies, male or female BALB/c albino mice weighing 18–22 g were obtained from the National Institute of Health in Islamabad. Animals were maintained in a controlled laboratory environment (12-h cycle of darkness and light, 20–25 °C), with access to food and drink ad libitum. All the animals were housed in the Department of Pharmacy's animal house at Abdul Wali Khan University Mardan. Animals were chosen to acclimatize to the lab setting before the start of the activities. This study was reviewed and approved by the ethical committee Abdul Wali Khan University Mardan with approval number EC/DP/AWKUM/231E.

### 2.3 Compounds

The tested compounds (Table 1) were synthesized using the previously described method [11].

**Table 1:** Chemical structure of compounds (1&2).

Codes	Structures and Names (IUPAC)
1	1-(3-hydroxyphenyl)-2-((5-(1-(6-methoxynaphthalen-2-yl) ethyl)-1,3,4-oxadiazol-2-yl)thio)ethan-1-one C_23_H_20_N_2_O_4_S
2	3,5-dibromo-4-methoxy-*N'*-(2-(6-methoxynaphthalen-2-yl)propanoyl)benzohydrazide C_22_H_20_Br_2_N_2_O_4_

### 2.4 Acute toxicity

BALB/c albino mice and pigeon models were employed in this research. Pigeons and mice (n = 7) of good health were chosen and given different doses. The animals were observed for any detrimental effects during the first 4 h, and the mortality rate was determined after 24 h. We examined the animals for signs of diarrhea, vomiting, and nausea. The clinical scores were also recorded during this research [12].

### 2.5 Antinociceptive activity

#### 2.5.1 Writhing test

A writing test was performed on BALB/c albino mice (20–22 g) to evaluate the antinociceptive effect of naproxen derivatives. The positive control group was given diclofenac sodium, the negative control group was treated with normal saline, and the test groups received naproxen derivatives. After 30 min of treatment, all groups of animals received an injection of 1 % acetic acid. After 5 min of acetic acid injection, abdominal writhing was observed for 10 min [12]. For the percent effect, the following formula was used:

$$Percent\;effect=100-\;\frac{No\;of\;writhes\;in\;Tested\;animals}{No\;of\;writhes\;in\;contol\;animals}\;X\;100$$

#### 2.5.2 Hot plate test

Food was withdrawn from animals in various groups for 2 h before the start of the studies. The hot plate surface temperature was 55 ± 0.2 °C. Animals were not allowed to persist on a hot plate for more than 15 s during their pre-testing stay. Naproxen derivatives were administered to test groups in varying doses *via* intraperitoneal (i.p) injection. Tramadol was administered to the positive control group, whereas normal saline was given to the negative control group. The groups to be treated were injected with NPDs at 1.25, 2.5, and 5 mg/kg doses. After 30 min of the above treatment, readings were obtained at 0, 30, 60, and 90 min. The duration spent on a hot plate without jumping, licking, or flicking the hind limb was recorded (the cut-off time was 30 s to avoid tissue injury) [12]. Naloxone injection was used to find the opioidergic mechanism. The following formula evaluated the percent effect:

$$Percent\;effect=\frac{\text{Latency}\;\text{time}\;\text{of}\;\text{test}-\text{latency}\;\text{time}\;\text{of}\;\text{control}}{\text{Cutt}\;\text{off}\;\text{time}-\;\text{Latency}\;\text{time}\;\text{of}\;\text{test}}\;X\;100$$

### 2.6 Anti-inflammatory activity

#### 2.6.1 Carrageenan-induces inflammation

To induce paw edema, a 100 L freshly prepared solution having 1 % carrageenan in distilled water was injected into the right paw of each rat in each group. The positive control group was administered diclofenac sodium, whereas the negative control group was given normal saline. Thirty minutes before the carrageenan injection, the test groups received intraperitoneal injections of various naproxen derivatives. The paw volume was measured at 0 h before the carrageenan injection, 1, 2, 3 and 4. The percent anti-inflammatory effect was calculated using the following formula [13].

$$Percent\;effect=\frac{\text{Edema}\;\text{of}\;\text{test}-\text{edema}\;\text{of}\;\text{control}}{\text{edema}\;\text{of}\;\text{control}}\;X\;100$$

#### 2.6.2 Bradykinin induces inflammation

The test compounds were administered 30 min before the bradykinin injection. Inject 0.1 mL bradykinin into a mouse hand paw. Readings were obtained at 0, 1, 2, 3, and 4 h after bradykinin injection. A plethysmometer was used to measure paw size changes [14].

#### 2.6.3 Prostaglandin induces inflammation

Paw edema was induced in all groups of BALB/c albino mice by injecting a 100 L freshly prepared solution of 1 % prostaglandin 2 in distilled water into the right paw. The positive control group was given diclofenac sodium. In contrast, the negative control group was given normal saline, and the test groups received naproxen derivatives at various doses as an intraperitoneal (IP) injection 30 min before the prostaglandin injection. Paw thickness was assessed at 0 h, shortly before the prostaglandin injection, and then at 1, 2, 3, 4, and 24 h later. The difference between paw thickness at 0 h and paw thickness at corresponding hours was used to calculate an increase in paw thickness [15].

#### 2.6.4 Xylene induces inflammation

The topically applied 0.03 mL of xylene was to the right ear, interiorly, and posteriorly of BALB/c albino mice. The control ear was the left one. The positive control group was given diclofenac sodium, the negative control group was treated with normal saline, and the test groups were treated with naproxen derivatives 30 min before the xylene application. After 2 h, detach both ears from the deceased mice and weigh them. The weight of the untreated left ear was deducted from the weight of the xylene-treated right ear [[16], [17], [18]].

### 2.7 Molecular docking studies

The low-resolution crystal structure PDB ID 6COX [19] was retrieved from the PDB database to carry out molecular docking using MOE software. Water molecules that were too far away from the active site were first removed, and then hydrogen atoms were added to the protein structure. After that, charges were assigned to each atom based on the Amber99 force field [20]. ChemDraw software was used to draw structures of compounds, and all these structures were optimized in MOE software. An MDB database was generated, and the structures were saved in the MDB database in mdb format. Molecular docking was performed using the Triangle matcher algorithm and a scoring function of London dG. A total of 10 poses were allowed to be generated for each compound [21]. PyMol software was then used to generate the 3D interactions of compounds in complex with COX2 [22].

### 2.8 MD simulation

A molecular dynamics (MD) simulation was carried out to examine the stability of the NP derivatives. The antechamber module of the Amber-22 program was used to generate the topology and coordinates files [23]. The systems were solvated in a 10 Å TIP3P water box, and then each system was neutralized with the addition of Na + ions. In this work, the protein force field ff19SB was employed for protein while the generalized AMBER force field (GAFF) was used for the ligands [24]. A Langevin thermostat was used to keep the temperature constant at 300 K, and a Berendsen barostat was used to keep the pressure constant at 1.0 bar. Each system was optimized using a two-step energy minimization process that involved conjugate gradient and steepest descent algorithms [25]. We employed the AMBER-22 Particle Mesh Ewald (PME) method to calculate the long-range electrostatic interaction. The GPU version of AMBER-22 was used to perform 20ns MD simulation of the two complexes such as PD1/COX2 and PD2/COX2. The CPPTRAJ module of the AMBER-22 program was used to analyze the trajectory data from each simulated system. Finally, Origin v2022 was used for graphical representation [26].

### 2.9 Statistical analysis

The results were presented as mean ± SEM (standard error of the mean) of different animals. The ANOVA was followed by post hoc Dunnett's multiple comparisons tests using GraphPad prism 9.5.0 (730). The effect was considered to be significant at the P < 0.05 level.

## 3 Results

### 3.1 Acute toxicity study (mice model)

The NPDs were found safe in experimental animals at tested doses of 10 and 15 mg/kg. The detail of clinical scoring is presented in Table 2.

**Table 2:** Acute toxicity studies of NPDs in mice.

NPDs	Dose mg/kg	Muscle Relaxation	Faces	Feeding	Urination	Drinking	Behavior	Mortality
1	10	–	Less	–	Less	Less	Disturb	–
2	10	–	Less	–	Less	Less	Normal	–
1	15	–	+	–	+	+	Disturb	–
2	15	–	+	–	+	+	–	–

### 3.2 Acute toxicity (pigeon model)

Both NPDs were safe in the pigeon model at 15 mg/kg. Nausea, vomiting, and diarrhea were seen against higher doses of NPDs, as shown in Table 3.

**Table 3:** Acute toxicity studies of NPDs in pigeon model.

NPDs	Dose mg/kg	Nausea	Vomiting	Diarrhea	Mortality
1	15	+	+	–	–
2	15	+	–	+	–

### 3.3 Antinociceptive effect

#### 3.3.1 Writhing test

**Fig. 1:**
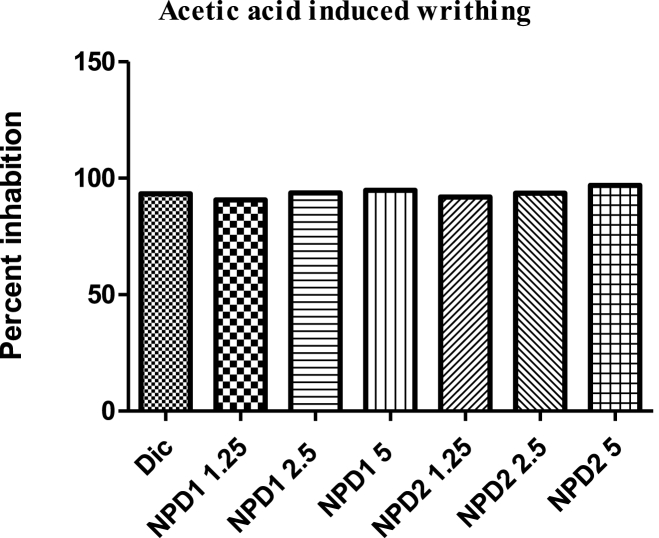
Percent analgesic effect of NPD.

Both NPDs showed significant (p < 0.001) antinociceptive effects in the acetic acid-induced writhing paradigm (Table 4). A done dependent effect was observed. The effect of NPD **2** was more than **1**. The maximum percent effect was 94.87 (Fig. 1).

**Table 4:** Acetic acid induced writhing test for NPDs.

NPDs	Dose	Writhing
DW	10 mL/kg	70.55 ± 0.97
Diclofenac	10 mg/kg	4.75 ± 0.99***
1	1.25	6.57 ± 1.00***
	2.5	4.45 ± 1.09***
	5	3.62 ± 0.91***
2	1.25	5.71 ± 0.98***
	2.5	4.55 ± 0.90***
	5	2.19 ± 1.12***

The data were presented as means ± SEM, and ANOVA was applied to find the significance level. *** = p < 0.001, ** = p < 0.01, * = p < 0.05.

#### 3.3.2 Hot plate method

**Fig. 2:**
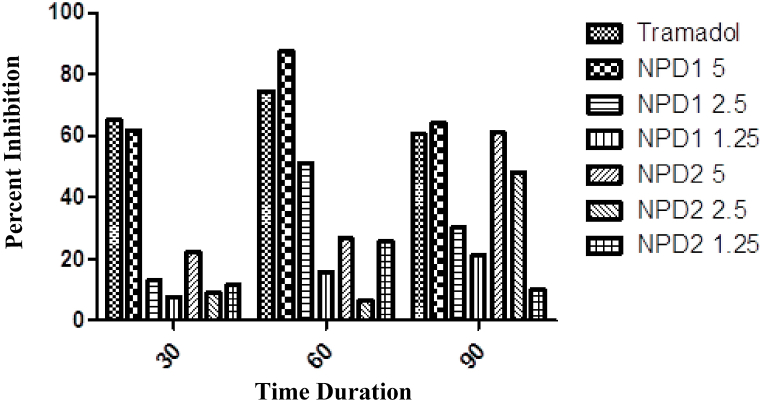
Hot plate study.

The antinociceptive effect of NPDs is shown in Table 5. The NPD **1** at the tested dose of 5 mg/kg enhanced the latency time after 60 min of injection, which remained significant (p < 0.001) up to the end of the experiment duration. The analgesic potential of NPD **1** was much better than NPD **2**. The maximum percent inhibition of NPD **1** was 87.53, as shown in Fig. 2.

**Table 5:** Analgesic effect of NPDs I hot plate model.

NPDs	Doses (mg/kg)	0 min	30 min	60 min	90 min
DW	10 mL/kg	6.18 ± 0.91	5.31 ± 0.94***	6.14 ± 0.92***	5.98 ± 0.95***
Tramadol	10	7.28 ± 0.94	18.12 ± 0.98***	20.21 ± 0.95***	17.53 ± 0.90***
1	5	7.76 ± 1.00	17.42 ± 1.01***	22.65 ± 1.03***	18.21 ± 1.05***
	2.5	6.7 ± 0.97	8.03 ± 0.99^ns^	15.79 ± 1.00**	11.73 ± 0.91**
	1.25	7.92 ± 0.94	6.81 ± 0.88^ns^	9.12 ± 1.11^ns^	10.00 ± 0.99*
2	5	8.12 ± 0.99	9.71 ± 0.99^ns^	11.20 ± 1.11*	17.61 ± 0.91***
	2.5	6.36 ± 0.95	7.12 ± 0.97^ns^	7.33 ± 1.00^ns^	15.14 ± 1.02**
	1.25	7.94 ± 1.04	7.56 ± 1.06^ns^	10.99 ± 1.76*	7.90 ± 0.99^ns^
Hot plate with naloxone					
1	2.5	7.83 ± 0.94	5.25 ± 0.90	7.63 ± 1.00	12.76 ± 0.95
2	2.5	6.54 ± 0.99	6.02 ± 0.92	9.87 ± 1.03	13.53 ± 1.00

The data were presented as means ± SEM, and ANOVA was applied to find the significance level. *** = p < 0.001, ** = p < 0.01, * = p < 0.05.

#### 3.3.3 Hot plate with naloxone

The naloxone injection significantly (p < 0.001) lowered the analgesic effect of NPD **1** as compared to NPD **2**. It indicates that NPD **1** has an association with the opioidergic pathway.

### 3.4 Anti-inflammatory activity

#### 3.4.1 Carrageenan paw edema

**Fig. 3:**
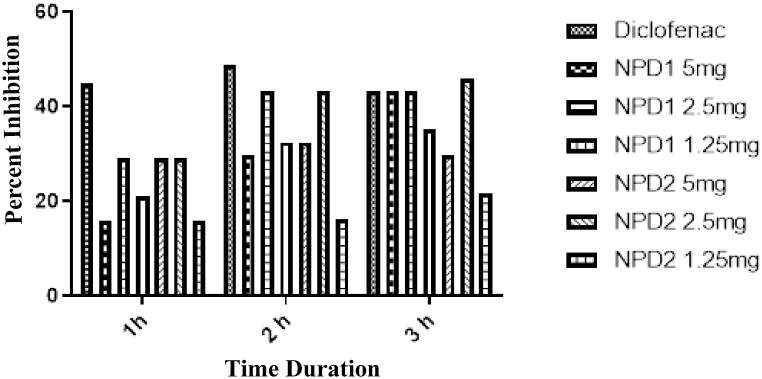
Carrageenan induce inflammation.

A dose-dependent and time-dependent anti-inflammatory effect was observed against both tested derivatives, as shown in Table 6. The NPD **1** proved more anti-inflammatory than NPD **2**. After 60 min, both compounds exhibited the maximum effect. The maximal percent inhibition by **1** and **2** was 43.24 (after 60 min) and 45.93 (after 90 min) as shown in Fig. 3.

**Table 6:** Anti-inflammatory effect of NPDs in Carrageenan/Bradykinin induce paw edema.

Carrageenan induces inflammation (Paw volume in mL)					
NPDs	Dose mg/kg	0 h	1 h	2 h	3 h
DW	10 mL/kg	0.36 ± 0.89	0.38 ± 0.90	0.37 ± 0.87	0.37 ± 0.86
Diclofenac	10 mg/kg	0.36 ± 0.91	0.21 ± 0.98***	0.19 ± 0.96***	0.21 ± 0.90***
1	5	0.33 ± 0.79	0.32 ± 1.00*	0.26 ± 0.92**	0.21 ± 1.02 *
	2.5	0.32 ± 0.78	0.27 ± 0.99***	0.21 ± 1.04***	0.21 ± 0.91***
	1.25	0.32 ± 0.90	0.30 ± 0.80*	0.25 ± 1.10**	0.24 ± 0.93**
2	5	0.30 ± 0.98	0.27 ± 0.87*	0.25 ± 0.87**	0.26 ± 1.09**
	2.5	0.24 ± 0.95	0.27 ± 0.99*	0.21 ± 0.88***	0.20 ± 1.04***
	1.25	0.28 ± 0.87	0.32 ± 0.94	0.31 ± 0.99	0.29 ± 1.02
Bradykinin inflammation (Paw volume in mL)					
1	2.5 mg	0.35 ± 1.00	0.30 ± 0.80*	0.26 ± 0.99**	0.26 ± 0.98**
2	2.5 mg	0.30 ± 0.97	0.28 ± 0.91***	0.24 ± 1.02**	0.22 ± 1.00***

The data were presented as means ± SEM, and ANOVA was applied to find the significance level. *** = p < 0.001, ** = p < 0.01, * = p < 0.05.

#### 3.4.2 Bradykinin induces edema

**Fig. 4:**
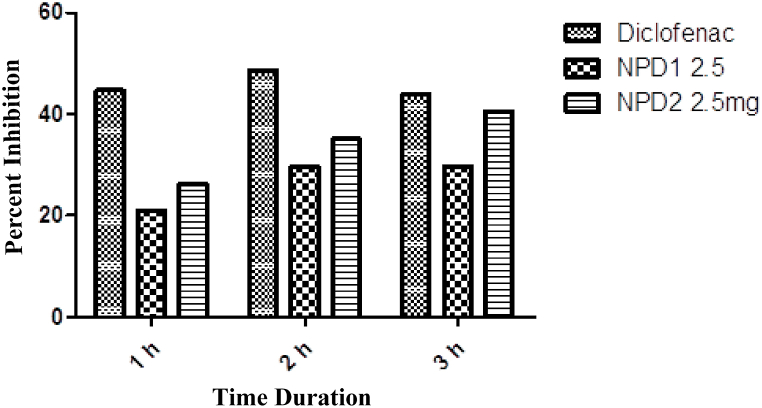
Bradykinin-induced inflammation.

In a bradykinin-induced inflammatory paradigm, both NPDs demonstrated significant (p < 0.001) anti-inflammatory activity (Table 6). Fig. 4 indicates that the maximal percent effect of NPD **1** and NPD **2** was 29.72 and 40.54, respectively.

#### 3.4.3 PGE 2-induced inflammation

**Fig. 5:**
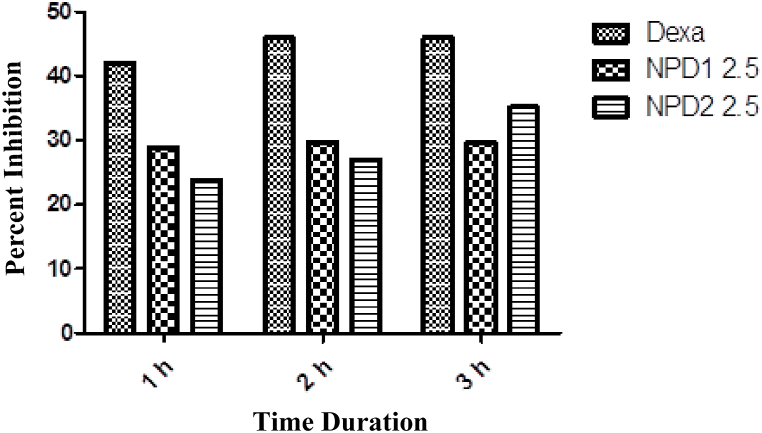
PGE2 induced inflammation.

In a prostaglandin-induced inflammatory model, both NPDs exhibited significant (p < 0.001) anti-inflammatory activity (Table 7). As indicated in Fig. 5, the maximal percent effect of NPD **1** and NPD **2** was 29.72 and 35.13.

**Table 7:** PGE 2-induced inflammation.

NPDs	Dose	0 h	1 h	2 h	3 h
DW	10 mL/kg	0.36 ± 0.89	0.38 ± 0.90	0.37 ± 0.87	0.37 ± 0.86
Dexamethasone	2 mg/kg	0.35 ± 0.87	0.22 ± 0.90***	0.20 ± 0.96***	0.20 ± 0.95***
1	2.5 mg	0.33 ± 1.01	0.27 ± 1.02**	0.26 ± 0.95**	0.26 ± 1.02**
2	2.5 mg	0.32 ± 1.03	0.29 ± 0.99**	0.27 ± 1.00**	0.24 ± 1.04***

The data were presented as means ± SEM, and ANOVA was applied to find the significance level. *** = p < 0.001, ** = p < 0.01, * = p < 0.05.

#### 3.4.4 Xylene induced inflammation

Compared to the non-treated ear, the xylene-induced inflammatory model substantially reduced ear edema, as shown in Table 8.

**Table 8:** Xylene-induced inflammation.

Xylene induced inflammation				
NPDs	Dose mg/kg	Right ear edema (mg)	Left ear edema (mg)	Percent effect
1	2.5	70.80	53.54	32.45
2	2.5	66.57	63.85	4.54

### 3.5 Docking analysis

**Fig. 6:**
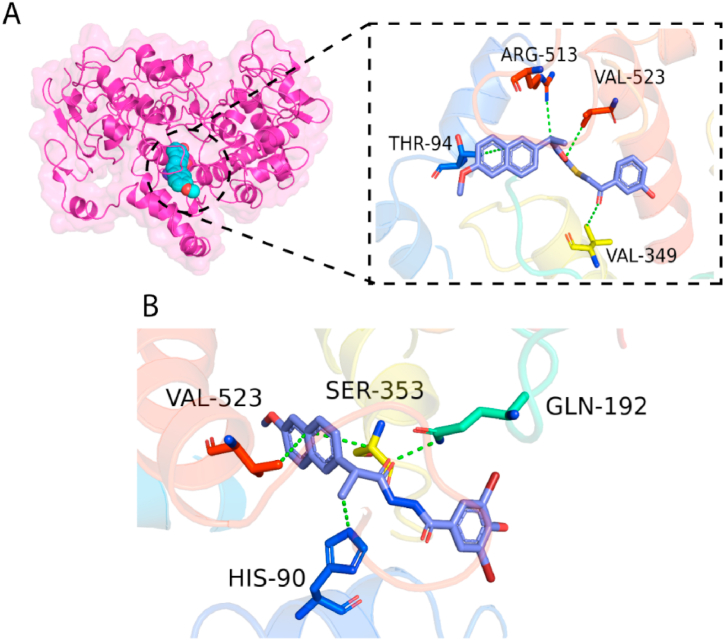
(A) 3D interactions of compound NPD-1 (B) compound NPD **2** with the active site residues of COX 2. Compounds are blue sticks, while the green dotted line represents the bond.

The docking score and interactions of drugs with receptors were computed following docking. A low docking score suggests that drugs have a strong inhibitory impact on the receptor. The docking score of compound NPD **1** was anticipated to be −7.32, whereas compound NPD **2**'s docking score was predicted to be −7.48. At distances of 3.4 and 3.7, compound NPD **1** formed two hydrogen bond acceptor contacts with active site residues such as Arg513 and Val349. Compound NPD **1** also formed two π-H contacts with Thr94 and Val523, which were separated by 3.8 and 3.7 nm, respectively. A similar contact pattern was seen for compound NPD **2**, which produced two H-bond acceptors and two pi-H interactions at distances of 2.9, 3.5, 3.6, and 3.8 with Gln192, His90, Ser353, and Val523. The interactions of molecules and docking score of NPD **1** and NPD **2** are shown in Table 9. Fig. 6 depicts the three-dimensional interactions of molecules with the receptor.

**Table 9:** Docking score and interactions of compounds NPDs with COX2.

Compound Codes	Interacting Residues	Interactions Type	Distance	Energy	Docking Score
NPD-1	ARG 513 VAL 349 THE 94 VAL 523	H-acceptor H-acceptor pi-H pi-H	3.46 3.76 3.85 3.73	−1.3 −0.5 −1.2 −0.8	−7.3246
NPD-2	GLN 192 HIS 90 SER 353 VAL 523	H-acceptor H-acceptor pi-H pi-H	2.93 3.51 3.64 3.80	−1.5 −0.5 −0.5 −0.9	−7.4830

### 3.6 MD simulation analysis

#### 3.6.1 Root mean Square deviation (RMSD) analysis

**Fig. 7:**
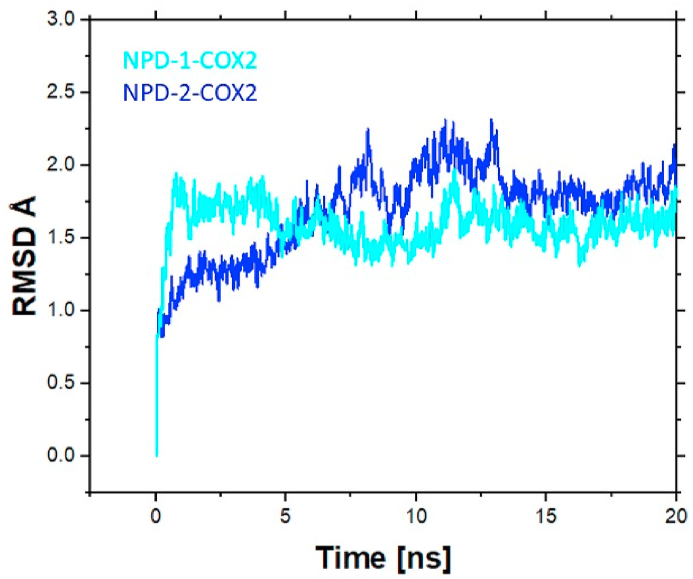
RMSD plot for NPD-1-COX2 (Cyan) and NPD-2-COX2 (Blue). Time in ns is present on the X-axis while the Y-axis displays the RMSD value.

To find the stability of compounds in complex with COX2 receptor RMSD as a function of time was calculated. RMSD analysis revealed that initially, the RMSD of the NPD-1-COX2 was high up to 1.9 Å but after 5ns the RMSD decreased and the system converged. Except for some minor deviations from 12 to 14ns, the RMSD of system NPD-1-COX2 remained highly stable till the end of the simulation. On the other hand, the RMSD of the NPD-2-COX2 complex revealed an unstable behavior during 7–14ns apart from these minor deviations no other major or minor deviations were seen during the entire 20ns MD simulation. The RMSD analysis revealed that both the compounds remained stable during the simulation which further supports the strong binding of both the compounds with the COX2 receptor. The average RMSD value was found to be 2 Å and 2.2 Å for NPD-1-COX2 and NPD-2-COX2 complexes respectively. Fig. 7 displays the RMSD plot for both systems.

#### 3.6.2 Root mean square fluctuation (RMSF) analysis

**Fig. 8:**
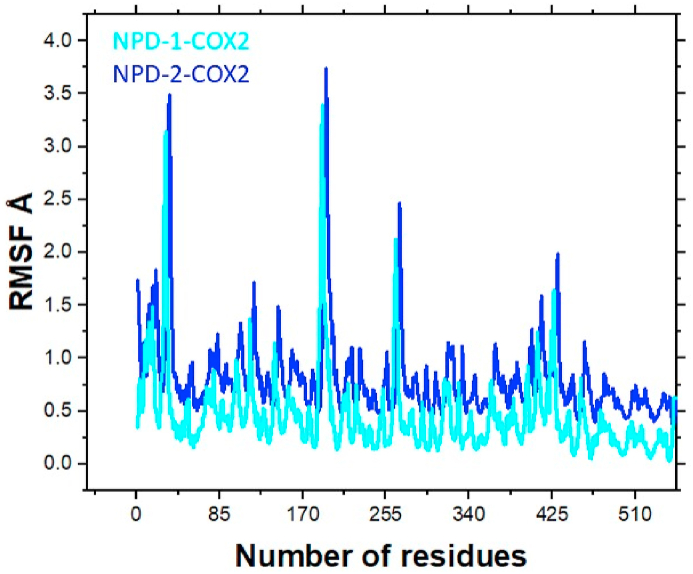
RMSF plot for NPD-1-COX2 (Cyan) and NPD-2-COX2 (Blue). The number of residues is present on the X-axis while the Y-axis displays the RMSF value.

The flexibility of each residue of the receptor can be determined by the RMSF analysis. The RMSF analysis reflects that among all the residues of the COX2, the active site residue revealed stable behavior. The residues that were far away from the active site revealed flexibility during the MD simulation. Almost a similar pattern of RMSF was observed for both complexes. The residues including His90, The94, Gln192, Val349, Ser353, Arg513, and Val523 revealed high stability during the MD simulation. Fig. 8 displays the RMSF plot for the two systems.

## 4 Discussion

Inflammation and pain are triggered by the same biological approach, cyclooxygenase (COX) intracellular signaling [27]. Prostanoids are synthesized using an enzyme known as cyclooxygenase (COX), which catalyzes the process. COX was discovered to have two distinct isoforms in the early 1990s. COX-1 is expressed as a "housekeeping" enzyme in most tissues. COX-2 can become more active in response to cytokines, lipopolysaccharides, and growth hormones [28]. All NSAIDs' universal principle of action is inhibiting the cyclooxygenase (COX) enzyme. The COX pathway converts arachidonic acid into prostaglandins (PGs) and thromboxanes (TX), promoting various physiological functions [29]. COX inhibitors are almost always required to alleviate or minimize these unpleasant effects. Whether these symptoms are pathological or non-pathological, they are treated in various ways, including COX inhibitors [30]. PGs are also responsible for multiple advantageous biological benefits, such as the stomach's mucosal lining and kidney protection. Therefore all the PG blockers will surely results in noxious effect, such as NSAIDs. Due to these noxious effects (also known as adverse drug reactions) patient compliance will decrease [31] leading to failure of therapy. The synthesis and testing of novel, efficacious, and safe derivatives are essential to address these issues with NSAIDs.

Naproxen is an important non-steroidal anti-inflammatory drug (NSAID) that is used to treat several inflammatory and painful conditions like rheumatoid arthritis, postoperative pain, migraine, headaches associated with tension, and pain linked with certain gynecological issues [32]. In practice, naproxen is also utilized in the levo (*S*) form; however, dextro (*R*) naproxen is not a pain reliever or anti-inflammatory. Therefore in the current research studies, all the NPDs are S-enantiomers instead of R-enantiomers. The pharmacological effects of naproxen are due to COX inhibition and decreased PG synthesis within the body. It has been associated with lower platelet aggregation due to decreased thromboxane production [33].

Compared to other NSAIDs (aspirin, indomethacin), naproxen has stronger gastrointestinal durability and has been used for many years due to its cardiovascular safety. It comes in two forms: naproxen sodium and free form. Since naproxen sodium dissolves quickly in stomach fluid, it has a more rapid effect, taking only 1 h to reach peak plasma concentration in contrast to naproxen, which takes 2 h [34].

The examined substances were also subjected to a molecular simulation in the current study, which revealed a significant interaction with the COX enzyme. The docking score of NPD **1** was −7.32, while the result of NPD **2** was −7.48, demonstrating a significant inhibitory effect. The current study intended to discover safe, efficacious, and cost-efficient naproxen derivatives. The present study determined both NPDs to be safe and effective. Surprisingly, the examined compounds significantly resolved the paw edema caused by numerous inflammatory mediators. NPDs considerably reduced inflammation, indicating a multi-mechanistic anti-inflammatory approach. In a carrageenan paw edema model, NPD **1** was a more potent anti-inflammatory than NPD **2**, and both NPDs reached their peak effect after 60 min. In the bradykinin inflammatory model, NPD **1** had the most significant effect after 60 min, while NPD **2** had the most excellent effect after 90 min. NPD **2** was more anti-inflammatory than NPD **1** in a bradykinin inflammatory model. In the PGE2 inflammatory model, NPD **2** had the highest anti-inflammatory potential after 90 min, whereas NPD **1** had the most increased activity after 60 min. NPD **2** was more anti-inflammatory than NPD **1**. In the xylene ear edema model, the most significant percent effect of the compounds NPD **1** and NPD **2** was 32.43 and 4.54, respectively. NPD **2** had significantly higher anti-inflammatory properties than NPD **1**.

The inhibition of acetic acid-induced writhing exhibited the peripheral analgesic effect. Acetic acid increases the permeability of the peritoneal membrane, causing abdominal muscular contraction as a pain indication. It was also found that acetic acid stimulated local pain receptors or the synthesis of prostaglandins. At 5 mg/kg, naproxen NPD **1** showed a dose-dependent reduction in writhing with a percent impact of 94.87. The chemical NPD **2** had a maximum percent writhing inhibition of 96 at a 5 mg/kg dose.

The current study shows that the NPDs we examined blocked the permeability of local pain receptors. The central analgesic potential of these NPDs was also evaluated utilizing a hot plate. The hot plate pain paradigm is a central analgesic model that induces pain by activating opioid receptors in the brain. Naloxone inhibited the increased latency time of NPDs, demonstrating that these NPDs have an agonist action. When compared to NPD **2**, NPD **1** produced significantly greater central analgesia. The naloxone injection significantly decreased the analgesic potential of NPD **1** while not affecting NPD **2**. The opioidergic pathway has been linked to NPD **1**. These *in vivo* and *silico* studies strongly support the tested compounds' analgesic and anti-inflammatory potentials. It is further suggested that more mechanistic study is required for the molecular mechanism of these compounds.

## 5 Conclusion

It is concluded that the tested NPDs are found safe in acute toxicity studies. The analgesic and anti-inflammatory effects of both naproxen derivatives were significant. The molecular docking analysis revealed that the naproxen derivatives significantly interacted with the COX2 enzyme. To evaluate the stability of naproxen derivatives in complex with COX2 receptor MD simulation was carried out. The MD simulation analysis revealed the high stability of the two naproxen derivatives upon binding to the COX2 receptor. This study confirmed the anti-inflammatory effects of both naproxen derivatives which can be used as inhibitors and can help reduce inflammatory-associated disorders.

## Data availability statement

The spectroscopic data and other physical data of compounds associated with this manuscript are available with the corresponding author upon request.

## Author's contributions

Conceptualization, N.M.; R.K.; F.S.; A.K, methodology; U.U.; A.W..; A.A.; U.K.; B.A, Software., K.M.K.; N.A.N, Validation N.A..; T.S.A, N, M Formal analysis, A.R.; N.M.; Investigation, N.K.; A.K.; Resources N.A, data curation, and original draft writing A.R. all authors contributed equally.

## Funding

None.

## Additional information

No additional information is available for this paper.

## CRediT authorship contribution statement

**Naveed Muhammad:** Formal analysis, Data curation. **Rashid Khan:** Formal analysis, Data curation. **Faiza Seraj:** Validation. **Abad Khan:** Formal analysis, Data curation. **Ubaid Ullah:** Methodology, Investigation. **Abdul Wadood:** Software, Formal analysis. **Amar Ajmal:** Investigation, Data curation. **Uzma:** Formal analysis. **Basharat Ali:** Investigation, Formal analysis. **Khalid Mohammed Khan:** Supervision, Formal analysis. **Noor Ul Ain Nawaz:** Methodology, Formal analysis. **Najla AlMasoud:** Investigation, Formal analysis. **Taghrid S. Alomar:** Investigation, Conceptualization. **Abdur Rauf:** Writing – original draft, Supervision, Investigation.

## Declaration of competing interest

The authors declare that they have no known competing financial interests or personal relationships that could have appeared to influence the work reported in this paper.
